# Integrating quantitative and qualitative methodologies for the assessment of health care systems: emergency medicine in post-conflict Serbia

**DOI:** 10.1186/1472-6963-5-14

**Published:** 2005-02-17

**Authors:** Brett D Nelson, Kerry Dierberg, Milena Šćepanović, Mihajlo Mitrović, Miloš Vuksanović, Ljiljana Milić, Michael J VanRooyen

**Affiliations:** 1 Center for International Emergency, Disaster and Refugee Studies Johns Hopkins Bloomberg School of Public Health: Department of International Health, Johns Hopkins University School of Medicine: Department of Emergency Medicine, 615 N. Wolfe Street, Baltimore, Maryland 21205, USA; 2 Emergency Center of the Clinical Center of Serbia, School of Medicine, Belgrade University, Pasterova 2, 11000 Belgrade, Republic of Serbia; 3 Division of International Health and Humanitarian Programs, Brigham and Women's Hospital: Department of Emergency Medicine, Harvard Medical School, 75 Francis Street Boston, Massachusetts 02115, USA

## Abstract

**Background:**

Due to the complexity of health system reform in the post-conflict, post-disaster, and development settings, attempts to restructure health services are fraught with pitfalls that are often unanticipated because of inadequate preliminary assessments. Our proposed Integrated Multimodal Assessment – combining quantitative and qualitative methodologies – may provide a more robust mechanism for identifying programmatic priorities and critical barriers for appropriate and sustainable health system interventions. The purpose of this study is to describe this novel multimodal assessment using emergency medicine in post-conflict Serbia as a model.

**Methods:**

Integrated quantitative and qualitative methodologies – system characterization and observation, focus group discussions, free-response questionnaires, and by-person factor analysis – were used to identify needs, problems, and potential barriers to the development of emergency medicine in Serbia. Participants included emergency and pre-hospital personnel from all emergency medical institutions in Belgrade.

**Results:**

Demographic data indicate a loosely ordered network of part-time emergency departments supported by 24-hour pre-hospital services and an academic emergency center. Focus groups and questionnaires reveal significant impediments to delivery of care and suggest development priorities. By-person factor analysis subsequently divides respondents into distinctive attitudinal types, compares participant opinions, and identifies programmatic priorities.

**Conclusions:**

By combining quantitative and qualitative methodologies, our Integrated Multimodal Assessment identified critical needs and barriers to emergency medicine development in Serbia and may serve as a model for future health system assessments in post-conflict, post-disaster, and development settings.

## Background

In June 1991, a series of civil wars began as the Socialist Federal Republic of Yugoslavia dissolved violently into the four independent republics of Slovenia, Croatia, Bosnia-Herzegovina, the Former Yugoslav Republic of Macedonia, and the semi-autonomous nations of Serbia and Montenegro. Kosovo remains a province of Serbia but under the administration of the international community. The events of the last decade in the Balkans have significantly impacted the health care system of Serbia. The nation and its health care system were devastated by international sanctions, soaring unemployment, political instability, near economic collapse, and the North Atlantic Treaty Organization (NATO) air campaign [1]. Consequently, the health care budget was cut dramatically, further limiting the system's capabilities of providing adequate health care. These difficult years resulted in a substantial fall in major health indices and left a crippled health system struggling to provide for the needs of its citizens [2].

A country's emergency medical services are a particularly important component of the health care system for they provide an essential front-line resource for trauma, medical emergencies, and those without other access to health care. Unfortunately, the system of emergency services in Serbia did not escape the devastation of the 1990s and continues to suffer from problems common throughout the health care system. Limited health care funding has resulted in a lack of necessary equipment, supplies, medications, personnel wages, and an economic infrastructure unable to support necessary health care reforms in the public and private sectors. Furthermore, the country has received little external support for health system reform, underscoring the need for focused assessments to best determine priorities for health care development.

There is a desperate need for reorganizing and restructuring emergency medical services throughout Serbia. To better understand the problems, needs, and obstacles to development, a team of investigators from Johns Hopkins University Schools of Medicine and Public Health collaborated with the Belgrade University Emergency Center to perform a multimodal assessment of emergency medicine in Serbia. Our goal in this study was to identify the most pressing needs and achievable goals, as perceived by emergency health care personnel, in order to ensure that development programs and available funds are appropriately directed [3].

Evaluating health care systems through the use of demographic data collection, surveys, or focus groups alone – as is often done in health system assessments – cannot adequately elucidate the intricacies of a country's health care system. In order to fully appreciate the complex dynamics of the health care system, it is necessary to perform a complete initial assessment; include strong local participation; identify and address significant barriers to change; and identify unique local needs and other cultural dimensions. To do this effectively, it is important to avoid simply introducing a "carbon copy" of a Western health care system that would further burden the system with inappropriate and unsustainable programs. We propose for the rapid assessment of health care services an Integrated Multimodal Assessment that uniquely combines diverse methodologies, whose individual effectiveness has been well described in the literature.

## Methods

This study used a multimodal approach to assess the strengths, needs, problems, and obstacles related to the development of emergency medical services in Belgrade. The four study modalities were 1) demographic information and observational data; 2) focus group discussions with 68 emergency medicine personnel; 3) individual, free-response questionnaires with 39 emergency medicine personnel; and 4) by-person factor analysis (or Q-methodology) of the attitudes and opinions of 36 emergency medicine personnel regarding the development of emergency medical services. Participants in the study were a non-probabilistic sampling of emergency medicine physicians, nurses, and department directors from all pre-hospital and hospital-based emergency medicine institutions in Belgrade. Many participants took part in more than one study modality. Data collection occurred during July-August 2002. The authors received institutional review board approval and the written informed consent of all participants.

### Characterization of emergency medical services

The capacity of emergency medical services in Serbia to provide for the needs of the country's citizens was characterized through health system data collection. Health system information was obtained through meetings with key officials in established health surveillance institutions such as the Emergency Center of the Clinical Center of Serbia; World Health Organization Belgrade office; Public Health Institute of Belgrade; Republic of Serbia Ministry of Health; European Agency for Reconstruction; Belgrade University, Institute of Social Medicine, Statistics and Health Research; as well as several non-governmental organizations with offices in Belgrade. Key officials were asked a series of quantitative, closed-response questions about the Serbian health care system and emergency medical services. Developed from a pilot health care assessment in Serbia the previous year, the questions focused on health system organization; patient population size and demographics; health care personnel; medical education and training; materials, supplies, and medications; and provider aptitude and morale [4].

### Focus group discussions

The two-fold purpose of the focus group discussions was 1) to better understand the needs, problems, and obstacles to system development as perceived by those involved directly in providing emergency medical services and 2) to collect a wide variety of opinions and attitudes for subsequent by-person factor analysis. The focus group discussions were held with 68 emergency medicine physicians, nurses, and directors through a non-probabilistic sample taken from the institute for pre-hospital emergency services, the Emergency Center of the Clinical Center of Serbia, and four of Belgrade's six community emergency departments. The discussions were conducted by the primary author in the Serbian language. The audio recordings of the 30- to 60-minute discussions were transcribed and translated into English by a native-English speaker fluent in Serbian (BDN) and a native-Serbian speaker fluent in English (MŠ). The translation was later verified by additional native-Serbian speaking co-authors fluent in English (MV, LM).

The investigative field team (BDN, KD, MŠ, MV, LM) summarized the final transcripts and extracted common themes and divisive statements for by-person factor analysis.

### Emergency medical provider free-response questionnaire

Free-response questionnaires enabled assessment of emergency health care needs and priorities for change as perceived by those most directly involved in patient care: the emergency health care providers. A non-probabilistic sample of 39 participants was obtained by open invitation, without compensation, through the director's office of each institution. As with the focus group discussions, the institute for pre-hospital emergency services, the Emergency Center of the Clinical Center of Serbia, and four of the city's six community emergency departments were represented. All subjects were administered a written Serbian-language questionnaire comprised of 10 free-response items (Table 1). The questions were developed through discussion with emergency medicine leaders at the University of Belgrade as well as through adaptation of earlier health care assessments conducted by the authors in the region. Each questionnaire was translated from Serbian to English by two bilingual translators (BDN, MŠ) and verified by additional authors (MV, LM) to ensure accuracy. Translated responses were coded and assessed for content by a researcher (KD) blinded to the demographics and specialty training of the respondents. More than one response was coded for each subject when necessary. Duplicate answers were only coded once. Illegible, blank, and off-subject answers were coded as missing data. Data were analyzed using SPSS 11.0 for Windows.

**Table 1:** Free-response questionnaire questions. Investigators developed and administered to participants a Serbian-language questionnaire containing the following open-ended questions.

**Free-response questionnaire questions**
• What functions best in emergency medicine in your country and why?
• How is the status of physicians in emergency medicine in comparison to that of physicians in other specialties?
• With regard to education and training, what are the obstacles to becoming a physician in the field of emergency medicine?
• What are the current problems in the system of emergency medicine? And what is being done to solve these problems?
• What are the priorities for improving the system of emergency medicine?
• What are the barriers to the future development of the system of emergency medicine?
• What types of training should be offered for the improvement of the system of emergency medicine?
• What are the strong and weak points of the training of physicians in the system of emergency medicine?
• What do you think would be the reaction of the health care community (physicians, nurses, administrators) to development of the system of emergency medicine?
• What do you think would be the response of the public and government to development of the system of emergency medicine?

### By-person factor analysis

By-person factor analysis, or Q-methodology, avoids many of the limitations of other modalities by allowing the grouping of participants based on their subjective responses to an issue while preventing investigator preconceptions from influencing the grouping structure. In a letter to *Nature *in 1935, physicist and psychologist William Stephenson introduced by-person factor analysis as a means for the scientific study of subjectivity [5]. The methodology uses a unique combination of qualitative and quantitative methods to subdivide a study population, evaluate the degree of consensus among the participants, and identify any discordant opinions. The process begins by creating a concourse, or collection, of opinions and perceptions toward the subject of interest – in this case, emergency medicine in Serbia. From this concourse, the investigators develop statements representing the spectrum of opinion and request that respondents assign each statement to a position within a quasi-normal grid distribution as to whether they completely disagree with, feel indifferently or ambivalently towards, or completely agree with the statement [6,7]. In our present study, a concourse was developed through the aforementioned focus groups and free-response questionnaires. Directly from this concourse, the field investigators (BDN, KD, MŠ, MV, LM) produced a Q-sample of 23 statements (Table 2), which was administered to 36 Belgrade emergency medicine physicians, nurses, and department directors. The participants were attained by open invitation through the director's office of each institution. Collected demographic information of participants included age, gender, and specialty training. Respondents were asked to sort the Q-sample using the condition of instruction, "Please sort these statements with respect to your opinion of the current system of emergency medicine in Serbia". Sorting involved the forced ranking of all statements within the grid of quasi-normal distribution. Statements with which the participant most strongly disagreed were placed toward one end of the grid (-3 agreement score). Toward the grid's other extreme (+3 agreement score) were placed statements with which the participant most strongly agreed. The participant placed statements that evoked ambivalence or neutrality near the center (0 agreement score) of the quasi-normal distribution. Factor analysis was then performed on the responses using PQMethod 2.10 [8] followed by manual factor rotation. This analysis leads to the subdivision of the study population into distinct "respondent types" (i.e. factors), which are clusters of participants grouped by their common opinions and perceptions. To define and characterize the resultant respondent types, all participants who loaded heavily on a respondent type (>60% concordance) were selected as respondent type "loaders". The participants who loaded heavily *and *specifically for a single respondent type (i.e. >60% concordance on the type of interest and <30% concordance on remaining types) were designated respondent type "definers" and closely reviewed to assist in characterizing each respondent type.

**Table 2:** Responses of emergency medicine physicians and administrators to Q-statements regarding emergency medicine in Serbia. Statements are listed from greatest to least participant consensus. With each statement, an averaged agreement score is calculated for all participants and for each identified respondent type. Scores represent the spectrum of participant agreement/disagreement (i.e. "strongly disagree" (-3), "ambivalent/neutral" (0), or "strongly agree" (+3) with the statement). To aid discussion of respondent types, summary labels ("Utilize", "Develop", and "Invest") characterize unique qualities of each respondent type. "Utilize" respondent type is most concerned with the poor utilization of emergency services. "Develop" respondent type advocates the further development of emergency medicine. "Invest" respondent type emphasizes the need for greater investment in emergency medicine.

**Q-statements **(listed in order of greatest consensus to least consensus)	**Averaged participant agreement score**	**Agreement scores of identified respondent types**		
		
		"Utilize" (5 loaders, 4 definers)	"Develop" (6 loaders, 3 definers)	"Invest" (4 loaders, 2 definers)
It is NOT necessary for patients to be seen by physicians in the field, but rather patients should be brought immediately to an emergency department for care.	**-2.0**	-2	-1	-3
Health management training for health care leaders is essential for the improvement of emergency medical services.	**-0.1**	-1	-1	-2
The public overuses ambulance services because there is no charge for the use of these services.	**0.6**	1	0	1
The first priority for the development of emergency medicine should be to improve the organization of emergency services.	**0.4**	2	1	0
Patients arriving to the emergency facility should be taken, according to their illness, directly to a specific specialty department.	**-0.4**	-1	-1	-1
Protocols should be developed to standardize the treatment of patients throughout Serbia.	**1.2**	0	3	-1
Emergency medicine should be taught as a required course during the last year of medical school.	**0.8**	-2	2	0
Emergency medicine in Serbia would function better if it were financed by the federal budget rather than by the social health insurance fund.	**0.0**	1	0	-1
Emergency medicine should be a separate specialty in which physicians are trained to exclusively practice emergency medicine.	**0.0**	1	2	-2
Primary health care providers in the health houses are sufficiently trained in the triage of emergent and non-emergent patients.	**-1.8**	-1	-2	-3
All institutions that provide emergency medical services should be open 24 hours a day.	**1.4**	0	2	1
A priority in the development of emergency medicine is to increase the number of appropriately equipped ambulances.	**-0.6**	-2	-3	1
There should be national guidelines to determine which illnesses/injuries should be treated at each type of health care facility.	**0.2**	2	0	1
Much of the burden on emergency health care providers is due to the time spent caring for non-emergent patients.	**1.3**	3	0	0
Continuing medical education should be required by law of all physicians working in emergency medicine.	**1.0**	0	1	2
There is poor coordination among the various specialties that provide emergency medical services.	**-0.3**	-1	1	-1
The public should be better educated about the level of care that each health care institution provides in order to properly use the available health care services.	**0.3**	3	0	0
The medical school is currently playing a sufficient role in the development of emergency medicine in Serbia.	**-1.6**	-3	-1	-1
There is poor cooperation between the emergency centers, clinical-hospital centers, pre-hospital emergency services, and health houses.	**0.6**	1	1	3
Physicians working in emergency medicine in Serbia need greater expertise and technical skills to provide an appropriate level of care.	**0.8**	-2	3	3
The problems in emergency medicine would be solved if there were money and equipment with which to work.	**0.4**	2	-2	2
There is an appropriate balance of theoretical and practical training for physicians in emergency medicine.	**-2.0**	-3	-3	-2
Radio communication does not function effectively between the ambulances and the medical institutions.	**-0.1**	0	-2	2

## Results

### Characterization of emergency medical services

Emergency medicine is not a recognized health care specialty in Serbia. In smaller communities, emergency services are provided by primary health stations (*zdravstvene stanice*) and primary health centers (*domovi zdravlja*). In the capital city of Belgrade, there exists a network of 6 part-time, hospital-based (*kliničko-bolnički centri*) emergency departments that are supported by around-the-clock pre-hospital emergency services (*hitna pomoć*) and the academic Emergency Center (*Urgentni centar*) at the Clinical Center of Serbia. Like many European countries, Serbia has adopted a largely Franco-German model of emergency medicine in which pre-hospital emergency services are provided in the field by physician-staffed ambulances [9]. Care is subsequently provided in-hospital by physicians of multiple medical specialties. Patients access emergency services through referral from Belgrade's 16 primary health centers, telephoning "94" for pre-hospital emergency services, or by personal referral to the emergency institutions. The academic Emergency Center at the Clinical Center of Serbia receives patients directly as well as by referral from community emergency departments that are closed or overwhelmed. During 2001, the Emergency Center received 130,877 patients.

### Focus group discussions

Through focus group discussions, the investigators were able to determine the opinions and perceptions of emergency service providers and administrators on the current emergency medical system in Serbia. Sixty-eight physicians participated in one of six focus group discussions (female: 39.7%; average work experience: 19.3 years; specialties: surgery (34%), internal medicine (29%), anesthesia (12%), general medicine (7%), emergency medicine (3%), other (15%)). Discussions were held at the institute for pre-hospital emergency services, the Emergency Center, and four hospital emergency departments.

The participants' comments from the focus group discussions are collectively summarized in Table 3. Most providers believe the lack of sufficient funding for emergency medical services is one of the greatest problems affecting emergency medicine in Serbia. Financial support for medications, medical supplies, modern equipment, employee salaries, and facility maintenance is extremely limited and restricts the capabilities of health care providers. Poor organization of emergency medical services, including the lack of government regulation, absence of uniform treatment protocols, improper system management, and poor triaging and routing of patients between facilities, also dominated the focus group discussions.

**Table 3:** Foremost problems of emergency medicine in Serbia and priorities for system development. A summary of the findings from focus group discussions with providers of emergency medical services.

**Results of focus group discussions with providers and administrators of emergency medical services**
**CITED PROBLEMS IN THE SYSTEM OF EMERGENCY MEDICINE IN SERBIA**

FINANCE:
≺ Inadequate financial resources for essential equipment, supplies, and medications
≺ Discouraged emergency medical service personnel due to meager salaries, difficult work conditions, and large workloads
≺ Inadequate number of properly equipped ambulances and functioning radio equipment
≺ Very few computers and no health information systems to track patient health records
ORGANIZATION:
≺ Need for federal regulation of emergency medical services
≺ Lack of sufficient protocols for the standardization of triage and treatment
≺ Inadequate coordination between the institutions providing emergency medical services
≺ Need for further development of emergency medicine as its own specialty
EDUCATION:
≺ Inadequate training in emergency medicine during medical school
≺ Insufficient practical training of emergency medical service providers
≺ Few opportunities for professional development and continuing education of emergency service providers
≺ Limited access to medical innovations through the internet, foreign professional journals, conferences, courses, and seminars
≺ Lack of health management training for leaders of health care institutions
≺ Need for public education about the emergency medical services system and how to properly utilize them
**SUGGESTED PRIORITIES FOR THE DEVELOPMENT OF EMERGENCY MEDICINE IN SERBIA**
FINANCE:
≺ Secure funding for essential medications, supplies, equipment, employee salaries, and maintenance of health care facilities
≺ Consider long-term sources of continuous funding for emergency services such as the government budget instead of the social health insurance fund
ORGANIZATION:
≺ Develop national protocols for the standardization of emergency triage and treatment
≺ Further develop emergency medicine as its own specialty
≺ Clearly define the responsibilities and emergency services of physicians in each health care facility
≺ Institute a system to promote better coordination between the primary health centers, the hospital emergency departments, and the Emergency Center
≺ Implement quality control measures for the delivery of emergency medical services
≺ Establish a health information system to facilitate the tracking of patients
EDUCATION:
≺ Introduce required continuing medical education supported by legislation that would provide health care professionals leave from work to attend this periodic training
≺ Provide health care professionals with access to continuing medical education through the internet, professional journals, conferences, seminars, and practical training
≺ Develop a fellowship program for emergency medicine physicians
≺ Increase the level of practical emergency medical experience provided in medical school and postgraduate training
≺ Implement training in BLS, ALS, and emergency triage for all health care providers
≺ Institute a health management training courses for leaders of health care institutions
≺ Educate the public regarding the level of emergent care that each health care institution provides and how to properly utilize the available health care services

In addition, physicians and medical directors report that their emergency medical system is in significant need of improved education and training programs. Suggested improvements include further training in emergency medicine during medical school, bedside training to develop practical skills, development of standardized treatment protocols, and access to continuing professional education.

### Emergency medical provider free-response questionnaire

Questionnaires were completed by 39 physicians at the institute for pre-hospital emergency services, the Emergency Center, and four hospital emergency departments (female: 41.0%; average work experience: 20.8 years; specialties: surgery (31%), internal medicine (31%), anesthesia (13%), general medicine (10%), emergency medicine (5%), other (10%)).

Respondents show several areas of high agreement – particularly remarkable considering the questionnaire's free-response format. The most frequently reported strength of Serbia's current health care system are the health care workers (69%). Worker enthusiasm (33%) and the collaboration of various medical specialties providing emergency services (21%) are most frequently cited as the greatest positive attributes of emergency service providers. In addition, 46% of respondents report that the various emergency medical service providers (especially the Emergency Center and the pre-hospital emergency services) are other strengths of emergency medical services in Serbia.

Thirty percent of the participants surveyed believe that lack of incentives to enter the specialty, difficulty of the work, and poor financial compensation are important barriers to becoming a physician in emergency medicine in Serbia. The poor reputation of physicians in emergency medicine relative to other physicians (reported by 46% of participants) may contribute to lower interest in specializing in emergency medicine. Poor organization (26%) and insufficient opportunities for professional development (15%) were also reported as major impediments to being an emergency service provider.

Participants were asked what they believed were major problems of emergency medical services in Serbia. Inadequate finances, medications, medical supplies, and modern medical equipment were cited by 54% of the emergency medicine personnel as being a major problem (Table 4). Of these respondents, 62% feel that they have sufficient technical skills but that insufficient medical equipment significantly restricts their capabilities as health care providers. Another 24% believe that the absence of adequate and continuous funding for emergency medicine is a critical problem. With improved funding they could obtain the necessary supplies, equipment, and medication they need, as well as improve employee salaries and facility maintenance. As a way to ensure a more reliable source of funding, 78% of the emergency medicine personnel surveyed emphasized the need for government financing of emergency medical services through the national budget rather than through the unpredictable social health insurance fund.

**Table 4:** Quantitative results of emergency medical provider questionnaires. Positive responses were calculated as a percentage of the number of providers that included the statement in their free response. More than one response was coded per subject when applicable. Italicized responses denote breakdown of individuals with the above response.

**Select questionnaire responses of emergency medical providers (n = 39)**	**Positive responses (%)**
CURRENT PROBLEMS IN EMERGENCY MEDICINE	

Organization	59

*An organized system doesn't exist*	35
*No government regulation*	*22*
*Lack of treatment/triage protocols*	*22*
*Poor coordination between health care facilities / lack of team work*	*17*
*Poor division of labor of those providing emergency services*	*17*
*Other*	*26*

Lack of supplies, equipment, and medications	54

*Insufficient equipment*	*62*
*Inadequate funding*	*24*
*Lack of optimal ambulances*	*5*
*Poor diagnostic & therapeutic procedures*	*5*

Training and education	36

Lack of incentives / difficult field of work / poor compensation	23

No answer / there is no system of emergency medicine	18

Pre-hospital emergency services / ambulance services	5

PRIORITIES FOR REFORM	

Organization	77

*Treatment protocols/guidelines*	*27*
*Improve coordination between health care facilities (team work) and within hospitals (between specialties)*	*27*
*Increase the number of physicians (personnel) trained in EM*	*17*
*Government regulation and organization*	*13*
*Legal regulation*	*10*
*Increase the number of beds*	*10*
*Establishment of EM as a separate specialty*	*7*
*Develop a computer database for tracking patients*	*3*
*Improve efficiency of system*	*3*

Supplies, equipment, and medication (improved diagnostics)	54

Training and education	36

Financing	33

Incentives / work conditions / compensation	21

BARRIERS TO FUTURE DEVELOPMENT	

Economics/resources	69

Organization	36

Political/government	26

Inadequate education and training	18

Reorganization of emergency medical services is a priority for development according to 77% of participants. Of these responses, poor coordination and team work between health care facilities (27%), the absence of treatment protocols (27%), insufficient numbers of emergency medical physicians (17%), and the lack of government regulation (13%) were the most frequently cited priorities for improving the organization of emergency medical services. Other concerns include lack of quality control measures, burden of caring for non-emergent cases, inadequate admission and triage areas, and insufficient hours of operation of hospital-based emergency departments.

Many emergency medicine personnel are also critical of the education and training of physicians in emergency medicine. Although 58% of those surveyed feel that physicians receive good theoretical training, 77% report that their educational system is in significant need of improved medical school and postgraduate training programs. Of the individuals advocating for improved education and training, 27% feel that further training in emergency medicine should be included in the medical school curriculum, and 53% believe that additional bedside training to develop practical skills is imperative. Furthermore, 56% of participants in this study express a need for better access to continuing professional education, including shared practical experiences and training between health care institutions. A large number of emergency medicine personnel (36%) stress the need for greater access to international journals, conferences, seminars, and the internet. Many (15%) also communicate a need for international collaboration and training programs between Serbia and foreign medical institutions.

Survey participants cite numerous obstacles to the development of emergency medical services in Serbia, including insufficient funding and resources (69%), poor organization of emergency services (36%), lack of governmental support (26%), and inadequate education and training (18%). Also vital to development efforts are the perceptions and attitudes of the people impacted by and involved in these efforts – the public, health care community, and government officials. A slight majority (62%) of individuals surveyed believe that the health care community will support further development of emergency medical services. However, only 46% and 41% of participants feel that the public and government, respectively, will respond positively to development.

### By-person factor analysis

Of the 36 emergency medicine personnel invited to participate in factor analysis, 33 individuals (91.7%) completed the exercise correctly (female: 45.4%; average work experience: 16.8 years; specialties: surgery (21%), internal medicine (27%), anesthesia (27%), general medicine (6%), emergency medicine (6%), other (12%)). The institute for pre-hospital emergency services, the Emergency Center, and two hospital emergency departments participated in this component of the study.

Factor analysis followed by manual factor rotation determined levels of agreement among the 33 participants and identified three unique types of respondents. Table 2 displays the averaged level of agreement and the level of agreement for each respondent type (the spectrum of agreement includes "I strongly disagree" (-3), "I feel ambivalent/neutral" (0), or "I strongly agree" (+3)). With statements written in the form of agreement, a majority of participants believe patients should be seen by physicians in the field (+2.0), protocols should be developed to standardize the treatment of patients throughout Serbia (+1.2), primary health care providers are not sufficiently trained in the triage of emergent and non-emergent patients (+1.8), emergency medical institutions should be open 24 hours a day (+1.4), much of the burden on emergency health care providers is due to time spent caring for non-emergent patients (+1.3), continuing medical education should be required by law of all physicians working in emergency medicine (+1.0), the medical school is not playing a significant role in developing emergency medicine in Serbia (+1.6), and there is not an appropriate balance of theoretical and practical training for physicians in emergency medicine (+2.0).

In addition to assessing levels of agreement among participants, by-person factor analysis allowed us to identify three different respondent types, each involving multiple respondents with diverse demographics but who share common and unique perspectives relative to the remaining participants. To aid discussion of the three respondent types, each was assigned a brief label: "Utilize", "Develop", and "Invest".

Respondent type "Utilize" was heavily loaded by a total of 5 respondents (15.2% of participants) and defined by 4 (12.1%). The principal concerns of the "Utilize" respondent type include the poor utilization of emergency medical services. These individuals believe that emergency personnel are well trained, educated, and prepared to address the emergent needs of the community. However, they consider their role ill-defined and misunderstood, leading to the ineffective use of their services by the public.

Respondent type "Develop" was characterized by 6 loaders (18.2%) and 3 definers (9.1%). These individuals support the further development of emergency medicine as an independent specialty in Serbia. They do not consider inadequate finances, supplies, or equipment to be the chief concern of the system of emergency medicine. Instead, the "Develop" respondent type encourages the development of standardized treatment guidelines, improved coordination between the specialties providing emergency care, and additional education and training in the specialty of emergency medicine.

The third respondent type, "Invest", included 4 loaders (12.1%) and 2 definers (6.1%). These individuals consider the system of emergency medicine appropriately organized but lacking adequate investment. They believe greater resources should be applied towards equipment and professional training and development. They are also concerned with an apparent lack of coordination between institutions providing primary health care and emergency services.

Additional respondent types were defined by single respondents, which prevented adequate characterization. However, the demographic data on each of these isolated respondent types were closely examined to assure that the individual was not in a unique position of authority that could disproportionately influence future health system reform. The specific responses of respondent types "Utilize", "Develop", and "Invest" to the 23 Q-statements are listed in Table 2.

## Discussion

The purpose of a rapid assessment is to provide organizations with timely and reliable information to aid targeted interventions. Many rapid assessment methodologies have been utilized, each with its own strengths and weaknesses. One of the most commonly used methodologies is the KAP (Knowledge, Attitudes, and Practices) survey, which is traditionally a standard questionnaire with pre-developed, closed-response questions [10]. Because of its ease in study development, administration, and analysis, a KAP survey can provide very rapid results for truly emergent situations. However, KAP surveys are limited in flexibility, community involvement, and internal validity. An alternative assessment method, Rapid Assessment Procedures (RAP), successfully addresses some of KAP's limitations through an ethnographic, participatory problem-solving process [11]. Nevertheless, while these methods may provide limited quantitative information for use in health sector assessment, they can lack sufficient detail to identify major barriers to system improvements.

The use of more detailed assessment tools – employing a combination of qualitative and quantitative methodologies – can more accurately characterize the country's health care needs and the significant political and cultural barriers to sustainable health care reform. Our proposed Integrated Multimodal Assessment utilizes a diverse approach for strong internal validity and by-person factor analysis to uniquely identify critical subpopulations. By understanding the concerns of subpopulations, more targeted interventions can be developed to directly address these concerns. A trial study evaluating primary health care in Serbia has demonstrated the advantages of using a combination of qualitative and quantitative tools for health system assessment [12]. Each of the integrated methodologies contributes its own strengths to the overall assessment, complementing shortcomings of the other methodologies. For example, while the questionnaires provide concise, quantifiable, and less equivocal responses, the focus group discussions allow investigators to seek clarification and have the unique benefit of participant interaction often leading to entirely novel ideas.

Out of the near collapse of the Serbian health care system comes the opportunity to establish a health system more effective than ever in meeting the needs of its citizens. Our focus group discussions, questionnaires, individual surveys, and by-person factor analysis reveal that most emergency medical physicians believe the greatest problems in their system are poor organization of emergency medical services; lack of essential funding, medical supplies, medication, and technical equipment; and inadequate education and training. Several respondents also share the idea that poor incentives for specializing in emergency medicine, difficulties of emergency service work, and inadequate compensation are significant barriers to advancing the field of emergency medicine in Serbia. Although many of these are problems ubiquitous to developing health systems, this study illuminates specific opportunities for emergency medicine providers, the Serbian government, and international institutions to work together to address these issues.

According to the majority of emergency medicine providers and administrators surveyed, of foremost priority is reorganization of emergency medical services. In order to begin addressing this need, several participants propose the development of triage and treatment protocols. To facilitate improved routing of patients within the hospitals and to the appropriate emergency health care facility, all health care providers, including those within the primary health centers, should receive formal training in triage. Several participants suggest that leadership development and management training should be provided to medical directors and leading health care providers to improve the organization within and between emergency health care centers. Public education programs should also be developed to increase awareness of the level of care that each health care institution provides so that the public can more properly utilize available health care services.

Study participants further reported that the attainment of necessary medications, supplies, and equipment should be a top priority in the development of emergency services. The professionals working in the health care system of Serbia are severely limited by a shortage of these resources and, therefore, are unable to apply their knowledge and skills for proper diagnosis and treatment. Obsolete medical equipment further constrain their diagnostic capabilities, and lack of basic medications and supplies impedes their treatment strategies.

This multimodal assessment also shows that the education and training of physicians in emergency medicine need further development. Elements of the educational system needing improvement include medical school and postgraduate bedside training programs; training between health care institutions; access to continuing professional education materials including international journals, conferences, seminars, and internet access; and international collaboration between Serbia and foreign medical institutions, including opportunities for Serbian physicians to gain international clinical experience. Several participants also suggest that all health care workers, including physicians working in the primary health centers, receive periodic training in Basic Life Support (BLS), Advanced Life Support (ALS), and emergency triage.

Further development of emergency medicine in Serbia will be complex and will face numerous challenges. Reform will require funding, political commitment, supportive legislation, a revised medical curriculum, multi-phasic implementation, and post-interventional evaluation. Respondents believe that the funding needed for development of emergency services represents a significant barrier to change, but that, however, it is not the universal solution.

Although a majority of the emergency medicine personnel surveyed feel that the health care community will support further development of emergency services, just under half of the participants expect similar support from the public and government. Additional studies specifically assessing the needs and concerns of the public and government are recommended.

The use of multiple assessment modalities and the resulting complexity of this study involve several limitations that require consideration. All participants in this study were invited to participate by the director's office at each hospital or clinic. The study participants were aware that their participation was entirely voluntary and that they would not receive compensation. Although the study did not provide an economic incentive for participation, it did provide the opportunity for those providers with strong opinions to openly voice their concerns and suggestions. As a result, the study population may not represent all emergency health care providers. While every emergency medicine institution in Belgrade participated, this study was limited to the pre-hospital and hospital-based institutions within the capital city (covering 14.9% of the population of Serbia (excluding Kosovo)). Nevertheless, the participants represent the breadth of medical specialties in Serbia, and they constitute a large number of the stakeholders involved in further development of emergency medicine.

## Conclusions

Despite the challenges, development of emergency services in Serbia can be accomplished through the dedication and commitment of providers. As a result of studies on emergency services in Kosovo, developmental programs similar to those suggested here have been successfully implemented to improve the training in and provision of Kosovo's emergency medical services [13]. Visiting experts currently travel to the province to provide didactic and bed-side training to physicians and other health care providers on the latest advances in emergency medicine, internal medicine, pediatrics, and several other fields of medicine. Similarly, a one-year fellowship program in emergency medicine and a leadership training program have been established at the University of Prishtina.

It is the hope of the authors that similar reforms will also be implemented to address the specific situation in the Republic of Serbia and that this multimodal assessment will assist by identifying the critical needs, barriers, and priorities for sustainable development. By utilizing both quantitative and qualitative methodologies, the authors submit that this Integrated Multimodal Assessment tool offers a more robust alternative to standard surveys, KAP studies, or the use of anecdotal information for quickly identifying priorities in health system reconstruction in the post-conflict, post-disaster, or development setting.

## List of abbreviations

KAP: knowledge, attitudes, and practices

RAP: rapid assessment procedures

## Competing interests

The authors declare that they have no competing interests.

## Authors' contributions

Each author has participated sufficiently in the work being reported to take public responsibility for the content. BDN, KD, and MVR conceived and designed the study and obtained research funding. MVR and MM supervised the completion of the study. BDN, KD, MS, MV, and LM undertook recruitment of participating centers and physicians. BDN, KD, and MS completed the data collection, statistical analysis, and first draft of the manuscript. All authors reviewed and contributed to the revision of the manuscript.

## Pre-publication history

The pre-publication history for this paper can be accessed here:


